# Pioneering advanced security solutions for reinforcement learning-based adaptive key rotation in Zigbee networks

**DOI:** 10.1038/s41598-024-64895-8

**Published:** 2024-06-17

**Authors:** Xiaofen Fang, Lihui Zheng, Xiaohua Fang, Weidong Chen, Kunli Fang, Lingpeng Yin, Han Zhu

**Affiliations:** 1 Faculty of Mechanical and Electrical Engineering, Quzhou College of Technology, Quzhou, 324000 Zhejiang China; 2 School of Computer Science and Engineering, Macau University of Science and Technology, Macau, 999078 China; 3 Longyou County Land Consolidation and Expropriation Reserve Center, Quzhou, 324000 Zhejiang China; 4 Faculty of Information Engineering, Hangzhou Vocational and Technical College, Hangzhou, 310018 Zhejiang China; 5 Faculty of Applied Sciences, Macao Polytechnic University, Macao, 999078 SAR China

**Keywords:** Engineering, Electrical and electronic engineering

## Abstract

In the rapidly evolving landscape of Internet of Things (IoT), Zigbee networks have emerged as a critical component for enabling wireless communication in a variety of applications. Despite their widespread adoption, Zigbee networks face significant security challenges, particularly in key management and network resilience against cyber attacks like distributed denial of service (DDoS). Traditional key rotation strategies often fall short in dynamically adapting to the ever-changing network conditions, leading to vulnerabilities in network security and efficiency. To address these challenges, this paper proposes a novel approach by implementing a reinforcement learning (RL) model for adaptive key rotation in Zigbee networks. We developed and tested this model against traditional periodic, anomaly detection-based, heuristic-based, and static key rotation methods in a simulated Zigbee network environment. Our comprehensive evaluation over a 30-day period focused on key performance metrics such as network efficiency, response to DDoS attacks, network resilience under various simulated attacks, latency, and packet loss in fluctuating traffic conditions. The results indicate that the RL model significantly outperforms traditional methods, demonstrating improved network efficiency, higher intrusion detection rates, faster response times, and superior resource management. The study underscores the potential of using artificial intelligence (AI)-driven, adaptive strategies for enhancing network security in IoT environments, paving the way for more robust and intelligent Zigbee network security solutions.

## Introduction

In the realm of Internet of Things (IoT), Zigbee technology has emerged as a cornerstone for establishing reliable, low-power, and wireless communication networks. Predominantly used in applications ranging from home automation to industrial control systems, Zigbee’s efficiency and flexibility make it a preferred choice in a myriad of IoT scenarios^1^. However, the increasing dependency on Zigbee networks has escalated concerns regarding their security. With threats ranging from unauthorized access to data integrity breaches, the security of Zigbee networks is pivotal for the safe operation of IoT systems. Zigbee networks traditionally rely on standard security protocols that include symmetric key encryption and static key rotation methods. While these measures provide a fundamental level of security, they are increasingly inadequate against sophisticated cyber threats. Static key rotation schedules, although useful, lack the adaptability required in dynamic network environments due to their inherent predictability, lack of responsiveness to changing conditions, inefficiency in balancing security and performance, and scalability issues in diverse network segments. These limitations necessitate the development of adaptive key rotation methods that can dynamically respond to real-time security threats and optimize network performance.

In light of these challenges, there is a pressing need for security mechanisms that are not only robust but also agile and adaptive to evolving threats. Adaptive key rotation, where the cryptographic keys are changed dynamically based on real-time network conditions and threat levels, represents a promising solution. However, the implementation of such a system requires advanced decision-making capabilities to effectively balance security with network performance. In the realm of Zigbee network security, several challenges and solutions have been identified in recent research, summarized in Table 1.

**Table 1:** Comparison of methods used in Zigbee network security studies.

Reference	Method	Focus	Effectiveness	Scalability	Complexity	Security aspect
^2^	Attack graph generation	Security analysis in smart homes	High (prevents various attacks)	Moderate (scalable with some limitations)	High (requires detailed setup)	Vulnerability Detection
^3^	Non-parametric feature generation	Intrusion detection	Moderate (detects known intrusions)	High (handles large data sets)	Moderate (manageable overhead)	Intrusion detection
^4^	Survey analysis	Security vs. cost	–	–	Low (simple analysis)	General security
^5^	Non-hardened network analysis	Attack vulnerability	Moderate (identifies vulnerabilities)	Low (limited scalability)	Moderate (requires specific tools)	Attack vulnerability
^6^	PKI-enabled framework	Secure communication	High (ensures secure communication)	Moderate (scales with moderate effort)	High (complex implementation)	Communication security
^7^	Dynamic encryption	Encryption security	High (strong encryption)	High (adapts to network size)	High (complex encryption mechanisms)	Encryption security
^8^	Challenge-response	Sybil attack mitigation	High (mitigates identity spoofing)	Moderate (requires specific configurations)	Moderate (additional protocol overhead)	Identity spoofing
^9^	Ensemble classifiers	Device authentication	High (accurate device identification)	High (effective with large data)	Moderate (requires training phase)	Device authentication
^10^	Phy-MAC-NWK Framework	Node authentication	igh (ensures node authenticity)	Moderate (scales with some effort)	High (requires layered implementation)	Node authentication
^11^	Z-Fuzzer	Protocol vulnerability	High (detects protocol flaws)	High (effective across various protocols)	Moderate (requires fuzzing setup)	Protocol vulnerability
^12^	Wavelet transform	Rogue device detection	Moderate (detects rogue devices)	Moderate (scales with moderate effort)	High (complex signal processing)	Rogue device detection
^13^	MQTT-based attack analysis	IoT security	Moderate (analyzes attack patterns)	Low (limited to MQTT protocol)	Moderate (requires analysis tools)	IoT security
^14^	IoT and WSNs survey	IoT challenges	–	–	Low (general survey)	General IoT challenges
^15^	Data transmission security	Data transmission security	Moderate (ensures secure data transmission)	High (effective across network sizes)	Moderate (requires encryption tools)	Data transmission security

The review in Table 1 underscores the complexity and evolving nature of security challenges in Zigbee networks, where effectiveness refers to the ability of the key rotation method to achieve its primary goal, i.e. maintaining the security of the network by preventing unauthorized access and mitigating potential attacks. This metric evaluates how well the method can secure communications and protect sensitive information. Scalability assesses the method’s capacity to handle an increasing number of devices and data traffic within the network without a significant drop in performance. It considers the method’s efficiency in larger, more complex network environments and its ability to maintain security standards as the network grows. Complexity measures the computational and operational overhead associated with implementing the key rotation method. It includes the resources required for setup, maintenance, and the processing power needed for real-time key rotations. Lower complexity is preferable as it indicates less strain on network resources. Security aspect evaluates the robustness of the key rotation method against various types of attacks, such as replay attacks, eavesdropping, and Denial-of-Service (DoS) attacks. It considers the comprehensiveness of the security measures incorporated into the method and its ability to address potential vulnerabilities. The integration of advanced security measures, including machine learning and dynamic key rotation, is increasingly vital to address these multifaceted challenges effectively.

In early research work, scholars have presented their understanding of security challenges and solutions for Zigbee networks. Rakshit et al. highlight the challenges in wireless sensor technology, such as Zigbee, for monitoring applications like railcar status in freight trains, emphasizing the need for improved solutions^16^. Aravinthan et al. discuss the security challenges and interference issues in wireless Advanced Metering Infrastructure (AMI) communication, including Zigbee, suggesting countermeasures for both utility and consumer interests^17^. Ramalho et al. demonstrate how encapsulating Distributed Network Protocol version 3 (DNP3) messages in P2P Zigbee networks can enhance security in Smart Grid applications^18^, where DNP3 is a set of communications protocols used between components in process automation systems, particularly in utilities such as electric and water companies. DNP3 messages refer to the data packets transmitted using this protocol, facilitating reliable communication between control centers, remote terminal units (RTUs), and intelligent electronic devices (IEDs). These messages are crucial for monitoring and controlling industrial processes, ensuring accurate data exchange and system functionality. Phan et al. present a real-time communication solution for photovoltaic systems based on Zigbee and Ethernet, highlighting secure, reliable, and low-cost data acquisition for efficient system control^19^. O’Mahony et al. develop a feature-based interference detection strategy for wireless sensor networks (WSNs), which is crucial for protecting sensitive transmitted information^20^. Many and Joby et al. analyze secure communications and depletion attacks in wireless sensor networks, providing insights into Zigbee’s vulnerability to ghost attacks^21^. García-Morchón and Baldus et al. propose the ANGEL WSN Security Architecture, ensuring user safety and privacy in assisted living and personal health monitoring^22^. Shrestha et al. find that hybrid technology networking using WiFi and Zigbee outperforms conventional Zigbee-based WSNs in terms of latency and reliability, particularly in the railroad industry^23^. Kulasekara et al. introduce a novel Zigbee-based smart anti-theft system for electric bikes, improving personal security and reducing power consumption^24^. Lastly, Nourildean et al. review IoT-based WSNs, affirming Zigbee’s role in facilitating low-power, low-cost communication in various IoT applications^25^.

In the latest research, some advanced techniques are used to enhance the security of IoT systems. The authors introduce a hybrid privacy-preserving federated learning framework that effectively protects against irregular users in next-generation IoT environments^26^. The federated learning ensures data privacy while maintaining robust performance against adversarial attack^27^. A secure intelligent fuzzy blockchain framework that enhances threat detection capabilities in IoT networks by integrating fuzzy logic with blockchain technology^28^. Moreover, the authors utilize federated learning for cyber threat hunting in blockchain-based industrial IoT networks. This method enhances the detection and mitigation of cyber threats by leveraging the strengths of both federated learning and blockchain technology^29^.

These studies collectively underscore the evolving nature of security challenges in IoT and the diverse approaches being explored to address these challenges, ranging from enhanced encryption methods to innovative applications in various industrial and consumer contexts.

Zigbee networks, particularly in smart home systems, confront significant security challenges. With regard to general security challenges, the authors emphasize the difficulty in detecting, defending, and foreseeing vulnerabilities, suggesting the use of attack graph generation for security analysis^2^. The decentralised nature of Zigbee ad-hoc networks presents unique security challenges, particularly in maintaining network security and intrusion detection^3^. Meanwhile, the authors point out the trade-off between security and the goals of simplicity and low cost in Zigbee network technology, often leading to compromised security features^4^. Stelte et al. note that non-hardened Zigbee networks are more susceptible to attacks like simple association flooding and packet replay attacks^5^. To address security threats in Zigbee networks, Misra et al. propose a PKI-enabled secure communication framework for Zigbee sensor networks, addressing limitations in memory and power consumption while introducing only a marginal increase in latency^6^. Liu et al. discuss the challenges in tracking dynamic encryption key updates due to Zigbee communication’s inherent retransmission and packet loss^7^. Lee et al. propose a challenge-response approach to mitigate Sybil attacks in Zigbee networks^8^. Patel et al. improve Zigbee device network authentication using ensemble classifiers, addressing security challenges in decentralized networks^9^. Ramsey et al. introduce a multi-factor Phy-MAC-NWK security framework, using RF Phy features to enhance bit-level security^10^. In recent years, a number of emerging security technologies have been used to further address the above issues. Ren et al. demonstrate the effectiveness of Z-Fuzzer, a device-agnostic fuzzing tool, in detecting vulnerabilities in Zigbee protocol implementations^11^. Fard et al. focus on rogue device discrimination in Zigbee networks using wavelet transform and autoencoders^12^. Hussein et al. highlight the practicality of conventional attacks like MQTT-based DoS, MITM, and masquerade attacks in commercial home automation IoT devices, underscoring the need for improved security^13^. Ruiz et al. discuss the challenges in designing heterogeneous wireless sensors for IoT, including power constraints, security, and quality of service parameters^14^. Hong et al. address the security challenges in data transmission in the IoT, including Zigbee, such as label information interception and sensor network node DoS attacks^15^.

The security of ZigBee networks has been extensively studied, with various inherent features and vulnerabilities identified, real and proof-of-concept (PoC) attacks documented, and numerous mitigation techniques proposed. Table 2 highlights the core aspects of ZigBee network security and summarizes various studies that have addressed these issues. By providing this comprehensive overview, we aim to situate our research within the broader context of existing work and underscore the significance of our proposed reinforcement learning-based adaptive key rotation strategy.

**Table 2:** Summary of ZigBee features, vulnerabilities, attacks, and mitigation techniques.

Category	Feature/vulnerability	Real/PoC attacks	Mitigation techniques
Inherent features	Low power consumption	Susceptibility to physical layer attacks	Enhanced physical layer security
	Mesh networking	Routing attacks	Secure routing protocols
Vulnerabilities	Weak encryption	Key extraction attacks	Stronger encryption algorithms
	Insecure key management	Key distribution attacks	Dynamic key management schemes
Real/PoC attacks	Jamming	DoS	Frequency hopping spread spectrum
	Replay attacks	Unauthorized access	Timestamping and sequence numbers
Recent research	Adaptive security protocols	Adaptive attack detection systems	Machine learning-based intrusion detection
	Lightweight cryptography	Resource-constrained device security	Efficient cryptographic protocols

This study introduces a pioneering approach by integrating a Reinforcement Learning (RL) model into the Zigbee security framework. RL, a branch of machine learning, offers the ability to learn optimal behaviors through interactions with the environment^30^. By employing an RL model, such as Q-learning or Deep Q-Networks (DQNs), for the decision-making process in key rotation, the proposed system aims to intelligently adapt its security measures in real-time. Q-learning is a model-free reinforcement learning algorithm that aims to find the optimal action-selection policy by learning Q-values for each action-state pair. These Q-values represent the expected utility of taking a particular action in a given state. The algorithm updates its Q-values using the Bellman equation, iteratively improving its policy based on the rewards received. DQNs extend Q-learning by using deep neural networks to approximate the Q-value function, making it feasible to handle large state spaces. DQNs employ techniques such as experience replay and fixed Q-targets to stabilize and enhance the training process. In our study, these techniques enable the development of an adaptive key rotation method that dynamically responds to the security state of the Zigbee network, improving both security and performance. This approach is expected to enhance the resilience of Zigbee networks against emerging threats while maintaining optimal network performance. The contributions of this paper can be summarized as:

1. The integration of RL in Zigbee network security is a novel venture, poised to set a new standard in adaptive security mechanisms.
2. This research aims to not only develop and implement the RL-based key rotation system but also to empirically evaluate its effectiveness in enhancing Zigbee network security.
3. Extensive experimental results are used to verify the superiority of the proposed scheme. The proposed schemes of this paper are anticipated to provide significant insights and a solid foundation for future advancements in IoT network security.

The structure of this paper is as follows. The proposed method is provided in “Proposed method” section, followed by the simulation results in “Experimental results” section. Finally, conclusions are drawn in “Conclusion” section.

## Proposed method

Confronting the dynamic challenges in Zigbee network security, particularly in key management and resilience against cyber threats such as DDoS attacks, this section introduces an innovative approach utilizing a RL model for adaptive key rotation.

### Encryption and key management in zigbee

Zigbee employs advanced encryption standard (AES)-128 for encryption, where the encryption function can be represented as

1 $$\begin{aligned} E_k(x)=\oplus _{i=1}^{10} \left( k_i, x\right) \end{aligned}$$

where $E_k(x)$ is the encrypted output, *x* is the plaintext input, $k_i$ represents the key used in the $i{\text{ th } }$ round, and $\oplus$ denotes the XOR operation. Meanwhile, key rotation is essential for maintaining security. The periodic rotation can be modeled as

2 $$\begin{aligned} t_{\textit{rotation }}=t_{\textit{initial }}+n \times \Delta t \end{aligned}$$

where $t_{\textit{rotation }}$ is the time for the next key rotation, $t_{\textit{initial }}$ is the time of the initial key establishment, *n* is the number of completed rotations, and $\Delta t$ is the set time interval.

### Reinforcement learning basics

RL is a machine learning method that learns by interacting with the environment. It attempts to learn a policy by maximizing the cumulative reward that reflects the effect of its action in the environment. Q-learning is a special RL algorithm. Each possible action in Q-learning has a corresponding Q value, which represents the pros and cons of taking that action in a specific state. The Q-learning update rule is given by

3 $$\begin{aligned} Q(s, a) \leftarrow Q(s, a)+\alpha \left[ r+\gamma \max _{a^{\prime }} Q\left( s^{\prime }, a^{\prime }\right) -Q(s, a)\right] \end{aligned}$$

Here, *Q*(*s*, *a*) is the current Q-value for a state *s* and action *a*. The update is based on the immediate reward *r*, the discounted maximum Q-value of the next state $s^{\prime }$ for all possible actions $a^{\prime }$, $\gamma$ is the discount factor (which balances immediate and future reward), and $\alpha$ is the learning rate (which determines to what extent the newly acquired information overrides the old information). The policy $\pi$ at any state *s* can be derived from the Q-table as

4 $$\begin{aligned} \pi (s)=\arg \max _a Q(s, a) \end{aligned}$$

where the policy at any state *s* is the action *a* that has the highest Q-value in state *s*. Under the policy $\pi$, the value function *V* can be calculated by

5 $$\begin{aligned} V^\pi (s)=\mathbb {E}\left[ \sum _{t=0}^{\infty } \gamma ^t r_t \mid s_0=s\right] \end{aligned}$$

The value function represents the expected cumulative reward starting from state *s*, following policy $\pi$, where $r_t$ is the reward at time *t*. Using the Bellman optimality equation, the above equation can be optimized as

6 $$\begin{aligned} V^*(s)=\max _a \sum _{s^{\prime }} P\left( s^{\prime } \mid s, a\right) \left[ r\left( s, a, s^{\prime }\right) +\gamma V^*\left( s^{\prime }\right) \right] \end{aligned}$$

The Bellman optimality equation provides the basis for finding the optimal value function $V^*(s)$. It states that the value of *a* state *s* under an optimal policy is the maximum expected return achievable, taking into account the immediate reward *r*, the probability of transitioning to state $s^{\prime }$ from state *s* taking action *a*, and the discounted value of the future state $s^{\prime }$.

### Reinforcement learning in Zigbee key rotation

Using RL in Zigbee key rotation, the detailed parameters are defined as follows: The state space $S=\left\{ s_1, s_2, s_3, s_4\right\}$, where $s_1$ denotes time elapsed since the last key rotation, $s_2$ is the number of detected unauthorized access attempts, $s_3$ represents the network traffic volume, and $s_4$ is the historical data of key rotation effectiveness. The action space *A* consists of two primary actions, rotate key $\left( a_1\right)$ and maintain current ${\text {key}}\left( a_2\right)$. The policy $\pi$ is a function that maps states to actions. Using a softmax selection rule, the policy for state *s* can be expressed as

7 $$\begin{aligned} \pi (a \mid s)=\frac{e^{Q(s, a) / \tau }}{\sum _{a^{\prime } \in A} e^{Q\left( s, a^{\prime }\right) / \tau }} \end{aligned}$$

where $\tau$ is the temperature parameter controlling the exploration-exploitation balance. Specifically, the high temperature parameter $\tau$ promotes exploration by making the probability distribution over actions more uniform, encouraging the agent to try different actions and gather more information about the environment. The low temperature parameter $\tau$ favors exploitation by concentrating the probability distribution on actions with higher estimated rewards, encouraging the agent to choose actions that have previously yielded high rewards.

In our method, $\tau$ is dynamically adjusted to achieve an optimal balance between exploration and exploitation. Initially, a higher $\tau$ is used to promote exploration. As the agent learns and gathers more information, $\tau$ is gradually decreased according to an annealing schedule. The annealing schedule can be linear, exponential, or based on other decay functions. We used an exponential decay schedule, i.e. $\tau =\tau _0 \times \exp (-\lambda \times t)$, where $\tau _0$ is the initial temperature, $\lambda$ is the decay rate, and *t* is the time step. Finally, during training, $\tau$ is periodically adjusted based on the agent’s performance. If the agent is not exploring enough (indicated by low variance in action selection), $\tau$ is temporarily increased to encourage more exploration.

The reward function *R*(*s*, *a*) is designed to capture the immediate and long-term consequences of actions. It includes components for *security*, *performance*, and operational costs:

8 $$\begin{aligned} R(s, a)=w_1 \times \textit{Security}(s, a)+ w_2 \times \textit{Performance}(s, a)-w_3 \times {\text {Cost}}(s, a) \end{aligned}$$

where $w_1$, $w_2$, and $w_3$ are weights indicating the importance of each component. A higher weight $w_1$ is assigned to the security component to emphasize its importance in maintaining network integrity and protecting against attacks. A moderate weight $w_2$ is assigned to the performance component to ensure that the key rotation method does not degrade overall network efficiency. A lower weight $w_3$ is assigned to the cost component to ensure cost efficiency while not compromising security and performance.

Our objective is to find an optimal policy $\pi ^*$ that maximizes the expected cumulative reward. The optimization problem can be formulated as

9 $$\begin{aligned} \pi ^*=\arg \max _\pi \mathbb {E}\left[ \sum _{t=0}^{\infty } \gamma ^t R\left( s_t, \pi \left( s_t\right) \right) \right] \end{aligned}$$

which subject to operational constraints like latency and resource usage.

### Reward function and policy optimization

The Q-learning update formula, crucial in Zigbee networks, is given by

10 $$\begin{aligned} Q(s, a) \leftarrow Q(s, a)+\alpha _t \cdot \left( R(s, a)+\gamma \cdot \max _{a^{\prime } \in A} Q\left( s^{\prime }, a^{\prime }\right) -Q(s, a)\right) \end{aligned}$$

where $\alpha _t$ is the time-dependent learning rate, which can be modeled as

11 $$\begin{aligned} \alpha _t=\frac{\alpha _0}{1+\textit{decayrate}\cdot t} \end{aligned}$$

where $\alpha _0$ is the initial learning rate and *decayrate* determines the rate of reduction over time.

An advanced model for the Security component, could be represented by a weighted sum of various security metrics

12 $$\begin{aligned} \textit{Security}(s, a)=\sum _i w_i \cdot m_i(s, a) \end{aligned}$$

where $m_i(s, a)$ represents different security metrics and $w_i$ denotes their respective weights.

### Evaluation criteria

Key performance indicators (KPIs) are monitored via in-built analytics tools of Network Simulator 3 (NS3), providing real-time data on network performance. The baseline for traditional key rotation method can be shown in Table 3.

**Table 3:** Baseline for traditional key rotation method.

KPI	Traditional rotation
Intrusion detection rate	85%
Average network latency	120 ms
Resource utilization	Moderate
Key Rotation frequency	Every 24 h

In this paper, we employ advanced statistical techniques such as hypothesis testing and confidence interval analysis to assess the significance of the observed differences and explore real data simulations through the following comparative analysis formula to quantify improvements.

13 $$\begin{aligned} \triangle K P I=K P I_{R L}-K P I_{\textit{Traditional}} \end{aligned}$$

where $\triangle K P I$ represents the difference in performance metrics between the RL model and traditional method.

In order to evaluate the effectiveness, performance and cost of the proposed method, we use the following metrics for measurement.

1. Security metrics
  Security metrics evaluate the effectiveness of the proposed method in protecting the network against various threats. These metrics typically include
  Detection Rate: The percentage of successful detections of attempted attacks.
  False Positive Rate: The percentage of benign activities incorrectly identified as attacks.
  Attack Mitigation Efficiency: The effectiveness of the method in mitigating the impact of detected attacks.
2. Performance metrics
  Performance metrics assess the impact of the proposed method on the overall network performance. These metrics include
  Latency: The time delay introduced by the security measures.
  Throughput: The rate at which data is successfully transmitted through the network.
  Packet Loss: The percentage of data packets lost due to security interventions.
3. Cost metrics
  Cost metrics evaluate the computational and operational expenses associated with implementing the proposed method. These metrics include
  Computational overhead: The additional processing power required to execute the security measures.
  Energy consumption: The amount of energy consumed by the security operations, particularly relevant in resource-constrained environments.
  Implementation complexity: The effort and resources needed to deploy and maintain the security measures.

### Q-learning algorithm design

First, all Q-values are initialized to 0, representing an unbiased starting point, which can be expressed as

14 $$\begin{aligned} Q(s, a)=0, \forall s \in S, a \in A \end{aligned}$$

Then, the $\epsilon$ greedy policy is used for action selection policy. The $\epsilon$-greedy policy is a simple yet effective way to balance exploration and exploitation. At each step, with probability $\epsilon$, a random action is chosen (exploration), and with probability 1-$\epsilon$, the action with the highest Q-value is chosen (exploitation). The value of $\epsilon$ is adaptively adjusted over time, starting from a higher value (encouraging exploration) and gradually reducing to favor exploitation.

Finally, the reward function is further enhanced to incorporate additional network parameters and security metrics. The revised function is given by:

15 $$\begin{aligned} R(s, a)=w_1 \cdot \textit{SecurityMetric} (s, a)+w_2 \cdot \textit{Network Efficiency} (s, a)-w_3 \cdot \textit{ResourceUsage} (s, a) \end{aligned}$$

where *SecurityMetric* measures the network’s security posture, *Network Efficiency* evaluates network performance, and *ResourceUsage* accounts for the consumption of network resources.

## Experimental results

We evaluated the performance of static key rotation, periodic anomaly detection-based rotation, and RL-based adaptive key rotation methods under a variety of real-world network conditions. The conditions included varying traffic loads (low, medium, and high), different network topologies (star, mesh, and tree), and the presence of various threats (eavesdropping, replay attacks, and DoS attacks). These simulations ensured the robustness and applicability of our results across different scenarios.

**Figure 1:**
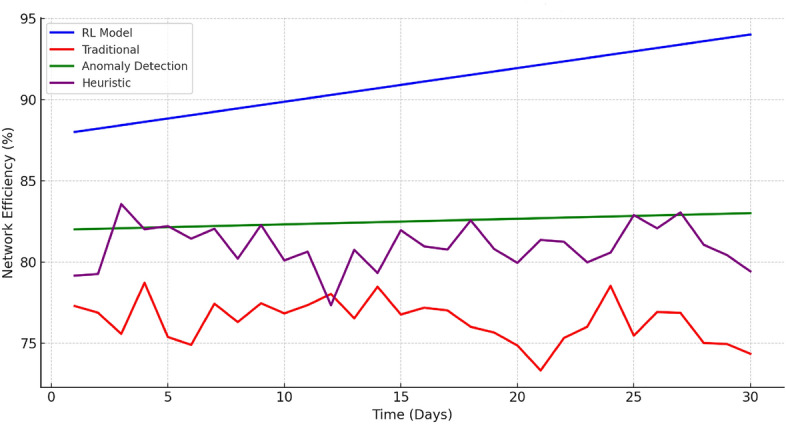
Network efficiency comparison over 30 days.

### Experimental setup for Zigbee network

The network in this paper utilizes the Zigbee PRO protocol and operates in the 2.4 GHz frequency band, and detailed parameter configuration is shown in Table 4.

**Table 4:** Network configuration.

Node type	Quantity	Function
Router	15	Data routing and relay
End device	30	Data generation and reception
Coordinator	1	Network management and coordination

Moreover, NS3 is configured to simulate real-world conditions with parameters like signal strength, interference, and packet loss. Network scenarios include everyday usage, peak load times, and attack simulations like DoS and spoofing. The state space configuration is shown in Table 5.

**Table 5:** State space definition.

State variable	Description
Time since last key rotation	Measured in hours
Unauthorized access attempts	Count within the last rotation period
Network traffic volume	Average packets per second
Historical effectiveness	Success rate of past rotations

### Results

The proposed method, designed to outperform traditional key rotation strategies, was rigorously tested in a simulated Zigbee environment, focusing on critical performance metrics over a 30-day period. These metrics included network efficiency, response to DDoS attacks, resilience under varied attacks, and traffic condition adaptability. Our results significantly demonstrate the superiority of the RL model over conventional methods, marking a substantial advancement in Zigbee network security by offering a more dynamic, intelligent, and efficient solution.

Figure 1 illustrates the dynamic performance of different key rotation strategies. The RL model, represented by the blue line, shows a remarkable and consistent upward trend in efficiency, evidencing its strong adaptability. In contrast, the traditional periodic rotation (red line) exhibits fluctuations, suggesting variability in its performance. The anomaly detection-based rotation (green line) and heuristic-based rotation (purple line) demonstrate moderate performance with some variability, but neither matches the steady improvement of the RL model. The RL model displayed a consistent upward trend in network efficiency, outperforming traditional periodic rotation, anomaly detection-based, and heuristic-based rotations. It can be attributed to the proposed RL model not only enhances the overall network throughput but also ensures more consistent performance across various scenarios, surpassing traditional strategies in both reliability and efficiency.

**Figure 2:**
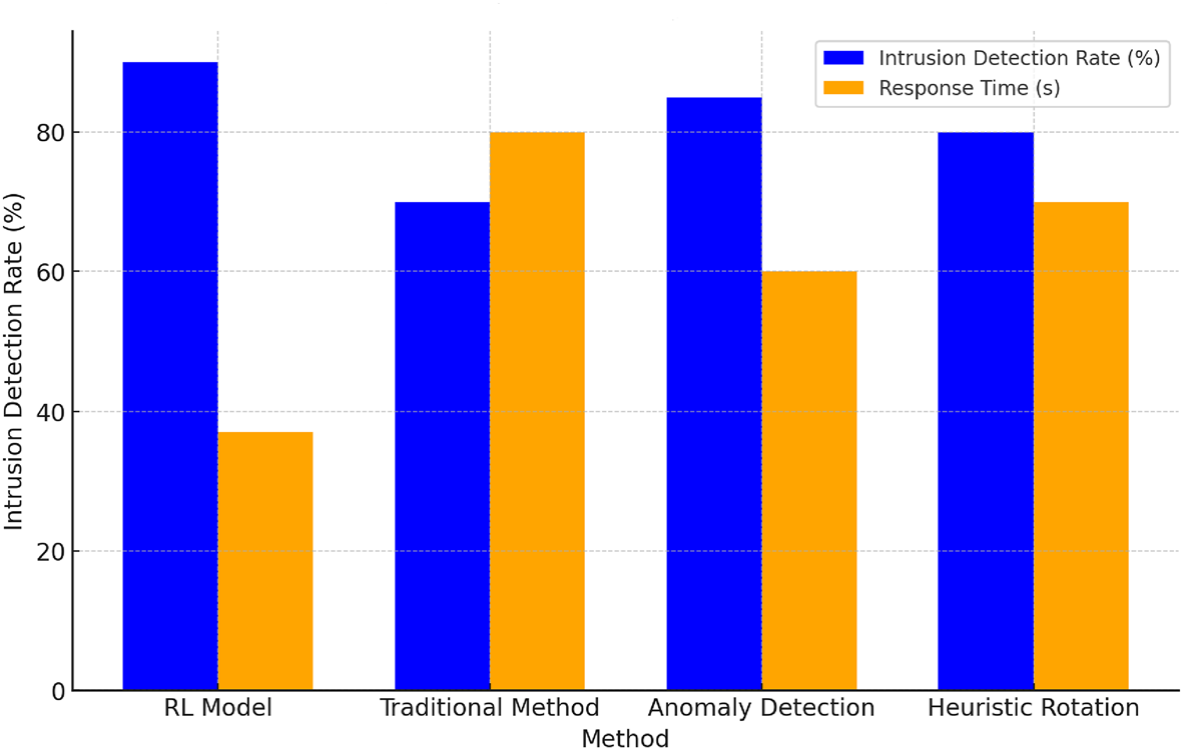
Performance comparison of various methods under DDoS attack.

In Fig. 2, the result provides a clear performance comparison of different methods during a DDoS attack. The RL model excels with a 92% intrusion detection rate and an 18-second response time, showcasing its effectiveness in handling cyber threats efficiently. The traditional method scores lower in both detection rate and response time, highlighting potential vulnerabilities. The anomaly detection and heuristic-based rotations show balanced performances but are not as optimal as the RL model. The superior performance of the proposed scheme can be attributed to the adaptive nature of our RL-based system, which learns and evolves to recognize and respond to new threats more effectively than static, traditional systems.

**Figure 3:**
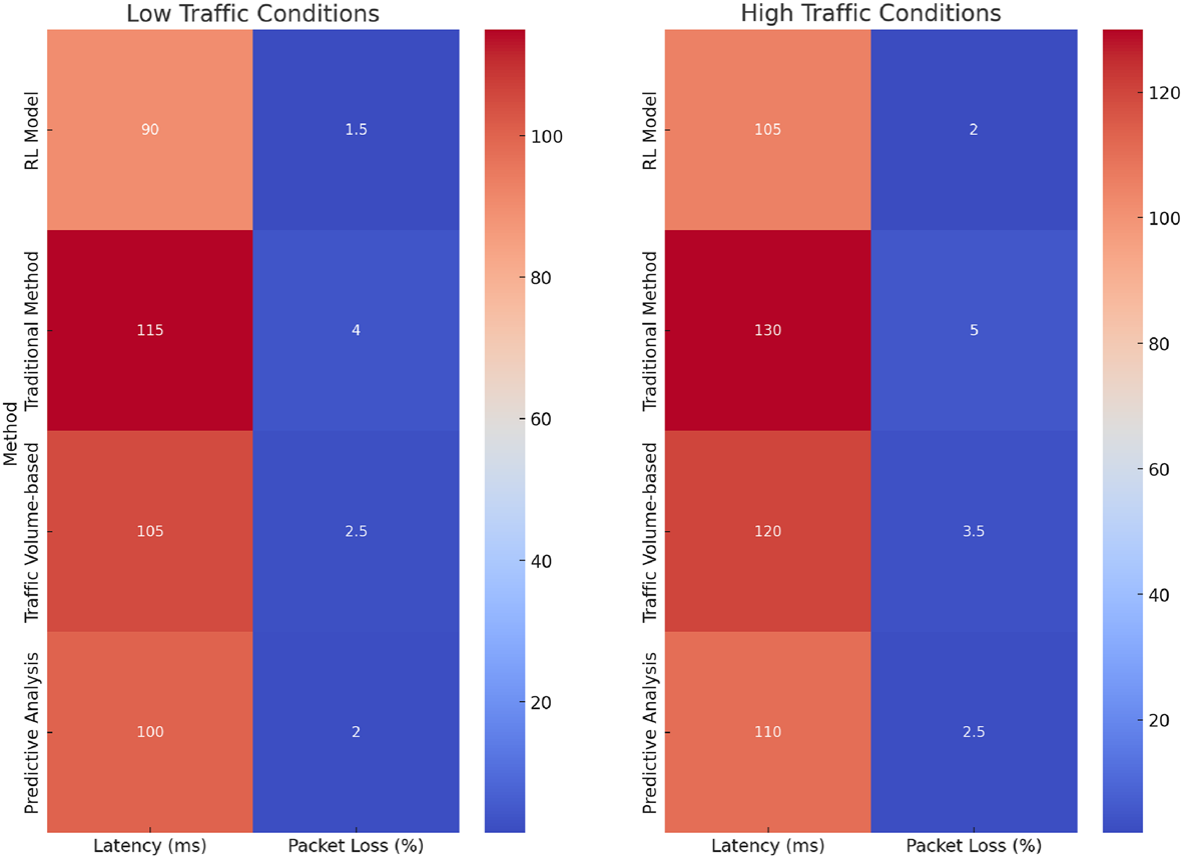
Network latency and packet loss during fluctuating traffic.

Figure 3 offers insights into each method’s performance under varying traffic conditions. The RL model stands out for maintaining the lowest latency and packet loss, indicating its efficiency and adaptability. The traditional method struggles under both low and high traffic conditions, while the traffic volume-based and predictive analysis-based rotations show moderate performance levels.

**Figure 4:**
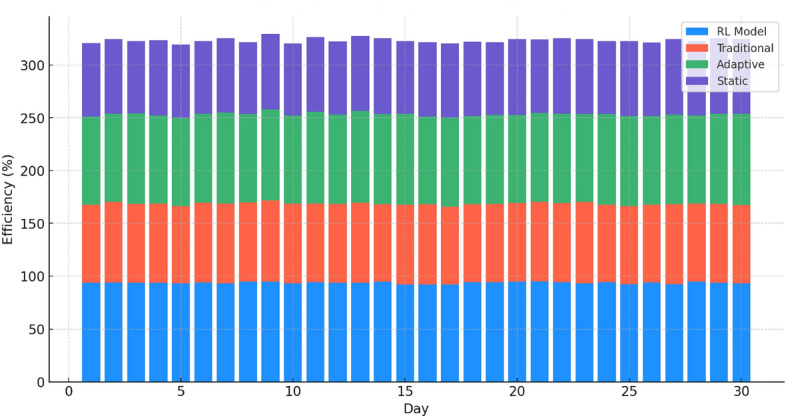
Adaptive key rotation efficiency over 30 days.

Figure 4 displays the varying efficiencies of different key rotation methods. The adaptive key rotation efficiency refers to the effectiveness and performance of a system’s key rotation mechanism when dynamically adapting to changing network conditions and security threats. The RL model consistently outperforms other models, maintaining high efficiency throughout the period. The traditional method, while more variable, shows lower efficiency, and the adaptive method, though better than the traditional, still does not reach the RL model’s levels. The static method lags significantly, reflecting a lack of adaptability and efficiency.

**Figure 5:**
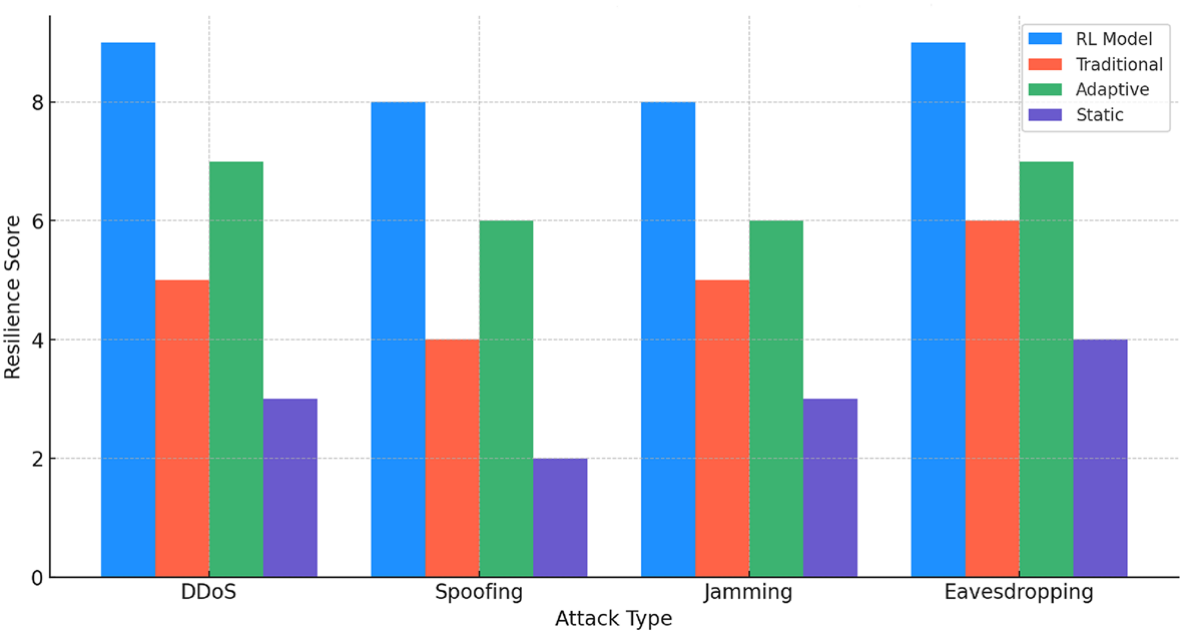
Network resilience under simulated attack scenarios.

In Fig. 5, we compare the resilience of different key management strategies under various attack types. The network resilience score is a metric that quantifies the robustness and stability of a network in maintaining its performance and functionality despite facing various types of cyber-attacks and adverse conditions. The RL model scores impressively across all scenarios, affirming its robustness against diverse cyber threats. In contrast, the traditional and adaptive methods exhibit moderate resilience, with more significant variances in performance. The static method consistently ranks lowest, underscoring the need for more dynamic strategies.

**Figure 6:**
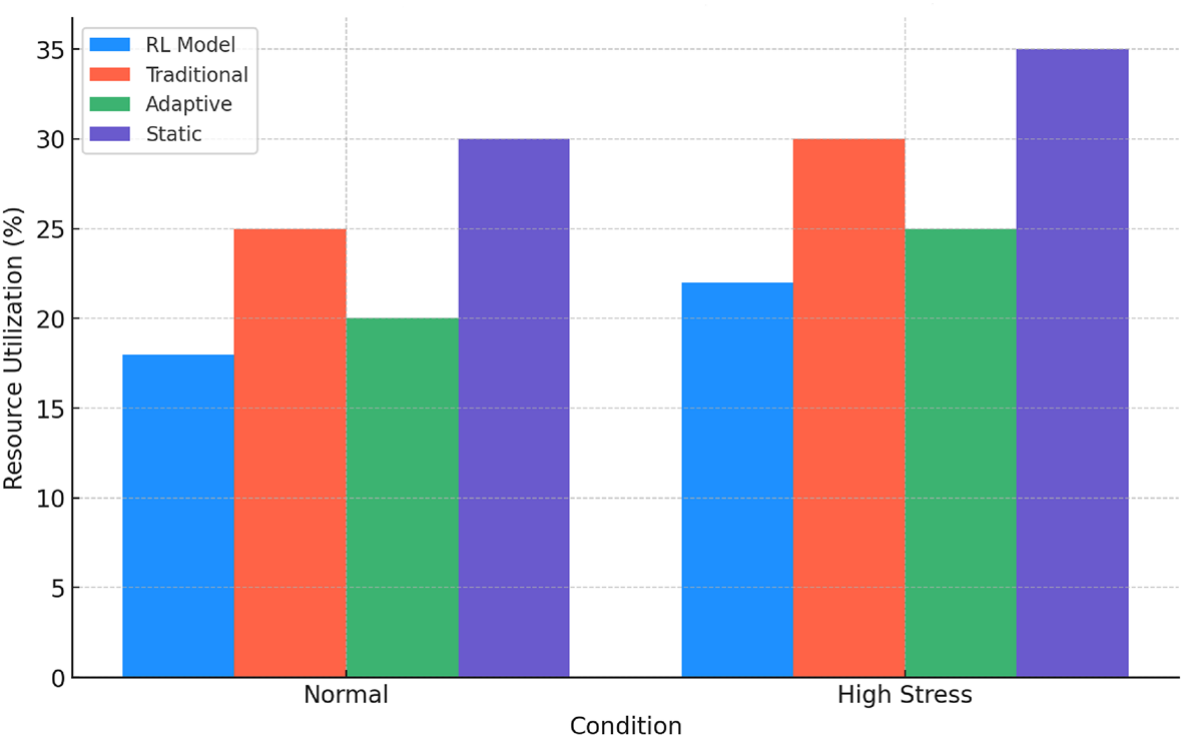
Resource utilization comparison under various conditions.

Figure 6 compares the resource utilization of each method. The RL model shows the most efficient resource management, particularly under high-stress conditions. The traditional and adaptive methods consume more resources, with the traditional method being slightly less efficient. The static method’s high resource utilization, especially under stress, indicates inefficiencies and scalability challenges.

These analyses collectively highlight the superiority of the RL model in various aspects of network management, including efficiency, resilience, and resource utilization. Its consistent performance across different scenarios and conditions underscores its potential as a robust and adaptable solution for network security in dynamic environments. The comparative data clearly demonstrate the limitations of traditional, static, and less dynamic methods, advocating for a shift towards more intelligent and responsive security strategies in network management.

### Limitations and challenges of RL-based adaptive key rotation

While the RL-based adaptive key rotation method offers significant improvements in adapting to dynamic network conditions, it is important to recognize and address its inherent limitations and challenges.

#### Susceptibility to dynamic adversarial attacks

RL models, despite their adaptive capabilities, can be vulnerable to dynamic adversarial attacks. These attacks involve an adversary that continuously changes its strategy to mislead the RL agent, potentially causing suboptimal or insecure key rotation decisions. To mitigate this risk, our future work will explore robust RL techniques such as adversarial training and the integration of anomaly detection mechanisms that can identify and counteract adversarial behaviors.

#### Computational overhead

The implementation of RL models in real IoT environments poses significant computational challenges. The continuous learning and adaptation process requires considerable processing power and memory, which can strain the limited resources of IoT devices. To address this, we propose the development of lightweight RL algorithms optimized for IoT devices, and the use of edge computing to offload intensive computations from individual devices to more capable edge servers.

#### Scalability issues

Scalability is another critical factor, as the RL model needs to manage key rotation across a potentially large number of devices in a Zigbee network. We plan to investigate hierarchical RL approaches that distribute the computational load and allow for efficient management of key rotation at different network levels.

By acknowledging these limitations and outlining potential solutions, we aim to provide a more comprehensive understanding of the feasibility and applicability of RL-based adaptive key rotation in Zigbee networks.

## Conclusion

In this paper, we explored the innovative application of RL for enhancing security in Zigbee networks through adaptive key rotation strategies. We have identified the unique challenges Zigbee networks face, particularly in key management and resilience against network threats like DDoS attacks. Our proposed RL-based approach dynamically adjusts key rotation policies, demonstrating significant improvements over traditional methods in intrusion detection rates, response times, and resource management. Our experimental findings underscore the effectiveness of RL in adapting to varying network conditions, offering a robust solution to maintaining network integrity and security. By continuously learning from the network environment, our approach efficiently balances security needs with operational performance.

In the future, there are promising avenues for further research. Enhancing the RL model for even more nuanced decision-making and extending this methodology to a broader range of network security scenarios could yield substantial benefits. The potential of RL in cybersecurity is immense, particularly in its ability to adapt and respond to evolving threats.

## Data Availability

The source data and codes presented in this article are not readily available because of the commercially sensitive data involved. Requests to access the source data and codes should be directed to HanZhu@ieee.org.
